# Development of a Multicellular 3D Tumor Model to Study Cellular Heterogeneity and Plasticity in NSCLC Tumor Microenvironment

**DOI:** 10.3389/fonc.2022.881207

**Published:** 2022-06-28

**Authors:** Leena Arora, Moyna Kalia, Suman Dasgupta, Navneet Singh, Anita K. Verma, Durba Pal

**Affiliations:** ^1^ Department of Biomedical Engineering, Indian Institute of Technology Ropar, Punjab, India; ^2^ Department of Molecular Biology & Biotechnology, Tezpur University, Assam, India; ^3^ Department of Pulmonary Medicine, Postgraduate Institute of Medical Education & Research (PGIMER), Chandigarh, India; ^4^ Department of Zoology, Kirori Mal College, University of Delhi, Delhi, India

**Keywords:** NSCLC, tumor microenvironment, multicellular 3D spheroids, cellular plasticity, tumor heterogeneity

## Abstract

Heterogeneity is a characteristic feature of solid tumors. Intra-tumor heterogeneity includes phenotypic diversity, epigenetic abnormalities, cell proliferation, and plasticity that eventually drives disease progression. Studying tumor heterogeneity in 2D culture is challenging as it cannot simulate the microenvironmental features, such as hypoxia, nutrient unavailability, and cell-ECM interactions. We propose the development of multicellular (tri-culture) 3D spheroids using a hanging drop method to study the non-tumorigenic (BEAS-2B) vs. tumorigenic NSCLC (A549/NCI-H460)cells’ interaction with lung fibroblasts (MRC-5) and monocytes (THP-1). Unlike the non-tumorigenic model, the tumorigenic 3D spheroids show significant induction of cell proliferation, hypoxia, pluripotency markers, notable activation of cancer-associated fibroblasts, and tumor-associated macrophages. CD68+ macrophages isolated from tumorigenic spheroids exhibited profound induction of phenotypic endothelial characteristics. The results are zebrafish tumor xenograft model and by using human patient samples. This multicellular 3D tumor model is a promising tool to study tumor-stroma interaction and cellular plasticity, targeting tumor heterogeneity, and facilitating cancer therapy success against NSCLC.

**Graphical Abstract:**
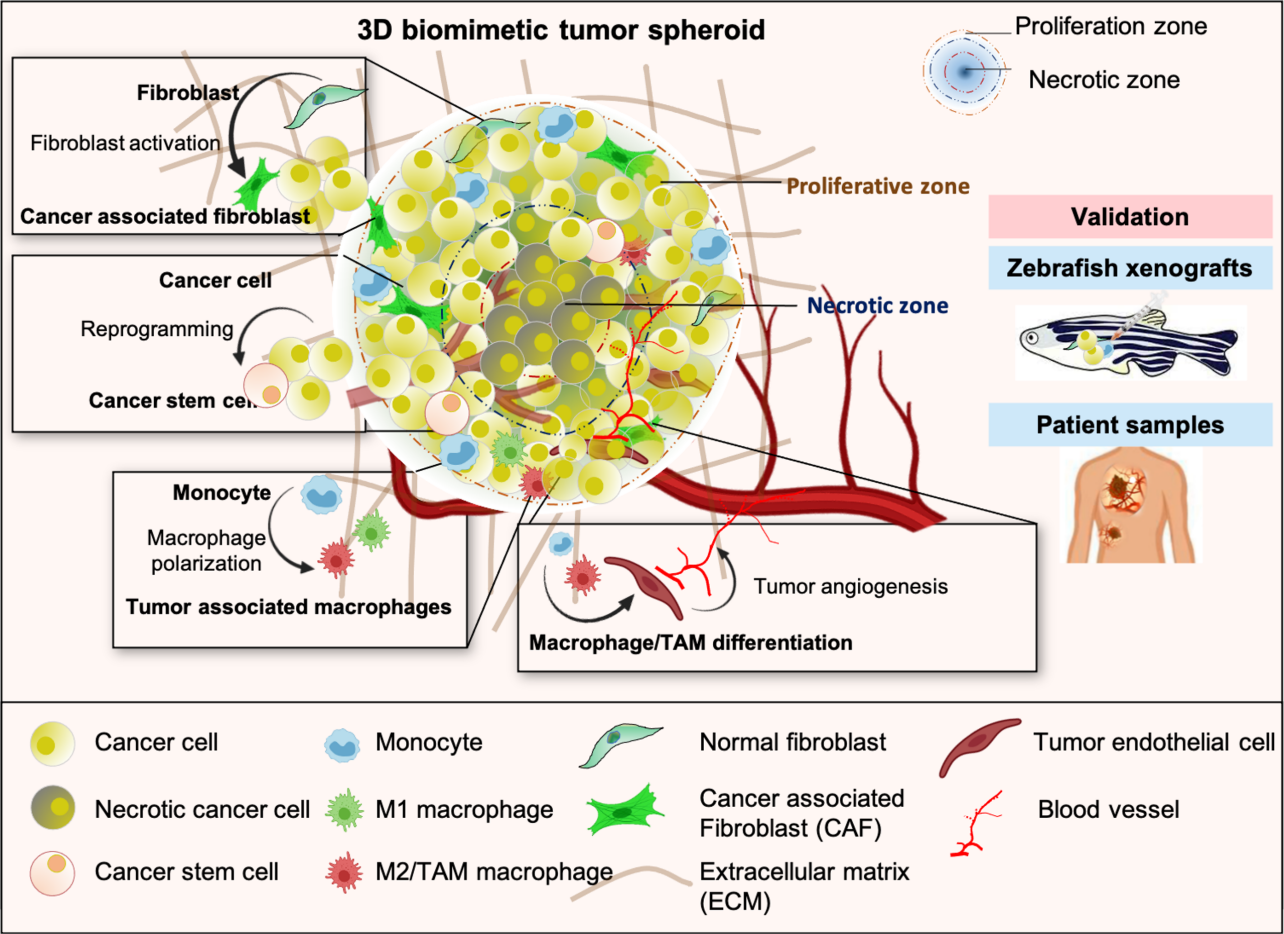
Schematic representation of multicellular 3D tumor spheroid with distinct proliferative and necrotic zones exhibiting key features of TME such as heterogeneity, cancer cell reprogramming and stemness, aggressive phenotype, alteration of stromal cells plasticity, and tumor angiogenesis. Results obtained from the study of 3D tumor spheroids were validated in the *in-vivo* experiments with zebrafish tumor xenograft model and lung cancer patients tissue samples.

## Introduction

Cellular heterogeneity is a vital feature of the solid tumors, influenced by the tumor microenvironment (TME). The origin of most of the cell types is unknown but attributes like pluripotency, differentiation, and trans-differentiation significantly contribute to cellular diversity (1, 2). Stromal and immune cells like fibroblasts, endothelial cells, monocytes, macrophages, dendritic cells, B cells, T cells, and their subsets reflect heterogeneity in the TME of lung, breast, renal, and other solid cancers (3–5). Uncovering the molecular signature and functional properties of each of these subtypes and the differentiation process is a critical requirement for developing advanced therapies against cancer. TME-associated cellular heterogeneity has challenged the current cancer regimens as the source of progenitor identities that created the phenotypic and functional differences is not explicit (6). It raises important questions like, (i) How does cellular phenotypic plasticity, an emerging hallmark of cancer (7), shape the TME? (ii) To what extent do cellular reprogramming, differentiation, and trans-differentiation processes contribute to tumor progression? Combining results from multiple experimental approaches will be essential to distinguish the relative contribution of varied processes, environmental cues, and cell-type differences on a tumor-promotive heterogeneous population. Also, studying this diverse cell population within human biopsies is challenging because of the substantial heterogeneity in tumor type, site, tumor stage, limitation of sample amount, and patient-specific variabilities (8). Thus, a reliable experimental model that could simulate the *in-vivo* tumor condition would be ideal for studying the tumor-stroma crosstalk and its involvement in disease pathophysiology. Therefore, 3D tumor models envisage an overall cellular heterogeneity and plasticity in a pathophysiological manner and are highly valued.

The spatiotemporal complexity of the TME necessitates the development of 3D multicellular models over 2D cultures. Multicellular 3D spheroids are self-assembled cell aggregates composed of two or more cells, usually 400-500μm in diameter, and can mimic the 3D conformational solid tumors and possess *in-vivo* physiological characteristics. Limited oxygen diffusion creates inner hypoxic zones and outer proliferative zone in 3D spheroids (9, 10). Interaction between tumor and stromal cells triggers the release of various cytokines and angiogenic growth factors, including vascular endothelial growth factors (VEGF), fibroblast growth factors (FGF), and platelet-derived growth factors (PDGF) that drives tumor complexity and cellular plasticity (11). Several manifestations achieve cellular plasticity during pathogenesis. A fully differentiated cell may reverse its course by reprogramming to an original progenitor or dedifferentiating back to its immediate progenitor. Alternatively, they may be enroute to the trans-differentiation process in which a differentiated cell switches its lineage to another cell type following an entirely different developmental program (7, 12). Considering this re-education, most heterotypic models studied either the activation of fibroblasts to cancer-associated fibroblasts (CAF), which have been shown to enhance the inflammatory environment, promote tumor progression, and drug resistance (13, 14). Some offer the activated processes of tumor angiogenesis, invasion, and metastasis in 3D spheroids involving mesenchymal stem cells and endothelial cells (15), or the alteration of immune cells, polarization of monocytes, or macrophages for inflammatory regulations (16, 17). Several other reports have focused on different chemotherapeutic or immune-targeted agents’ efficacy in targeting cancer cell death in 3D spheroids or addressing the plasticity of any stromal and immune cell components (18, 19). However, a 3D spheroid that potentially mimics the complex heterogeneity of TME and associated dynamic processes of trans-differentiation is of utmost need in the current scenario for the development of TME targeted cancer therapeutics.

Therefore, we developed a 3D multicellular tumor spheroid model to study tumor-stroma crosstalk and cellular plasticity. Crosstalk between tumor cells, fibroblasts, and monocytes alters gene expression profiles in fibroblasts and monocytes to their activated pathophysiological states. Moreover, activated fibroblasts in a 3D spheroid massively expressed ECM components such as stress fiber network-α-smooth muscle actin (α-SMA) and fibronectin; therefore, no additives or scaffolds mimicking ECM components are required to develop these multicellular spheroids. Interestingly, CD68+ macrophages in late-stage 3D tumor spheroids exhibit increased levels of endothelial markers, suggesting a possible myeloid lineage shift. Therefore, this multicellular 3D tumor model allows us to investigate the tumor-stroma interactions and cellular plasticity within tumor microenvironment.

## Results

### Development of Multicellular Tumorigenic and Non-Tumorigenic 3D Spheroids

**Figure 1:**
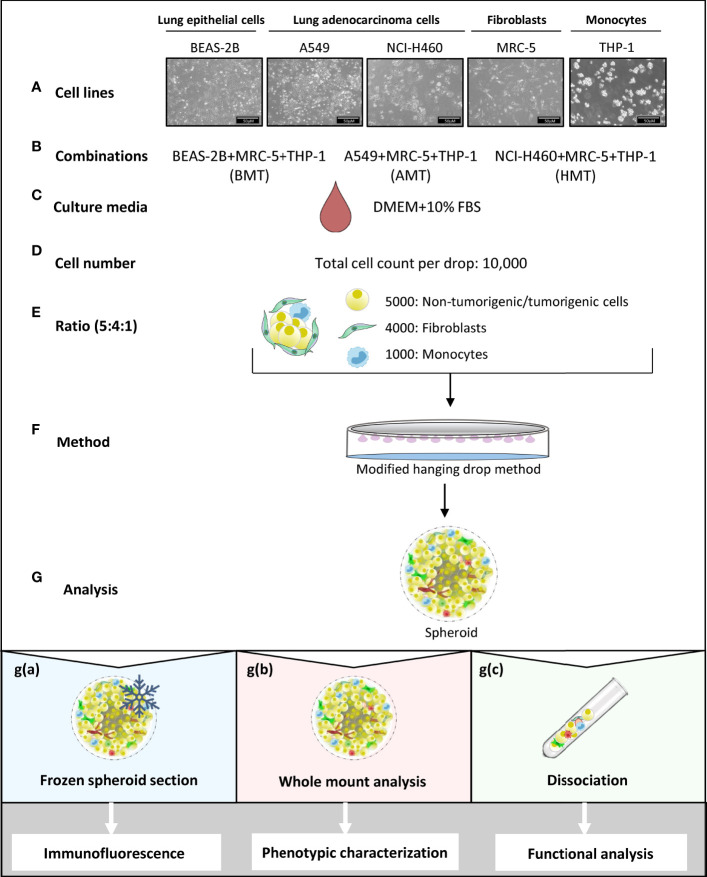
Schematic representation of multicellular 3D spheroid development protocol and their use in different experimental analysis. **(A–F)** Normal lung epithelial cells (BEAS-2B), lung adenocarcinoma cells (A549 and NCI-H460), MRC-5 fibroblasts, and THP-1 monocytes are used to prepare multicellular tumor spheroids (BMT, AMT, and HMT) in DMEM+10% FBS growth medium. After optimization, as detailed in the result section, 10,000 cells per 25 μl drop in a ratio of 5:4:1 (BEAS-2B/A549/NCI-H460: MRC-5: THP-1) are taken to prepare spheroids using hanging drop method. **(G)** Different techniques are used for spheroid analysis; **g(a)**. Spheroids frozen at low temperature (-80°C) were sectioned and stained with fluorescent-labeled antibodies for immunofluorescence analysis of cell proliferation, hypoxia, and cell plasticity in the spheroid microenvironment. **g(b)** Whole-mount analysis was performed for spheroid characterization (cell diameter, uniformity, cell viability), and functional analysis (gene expression and sprouting assays). **g(c)** Dissociation of multicellular spheroids by enzymatic digestion resulted in cell suspension, which was used for colony formation assay (cell proliferation), and flow cytometric analysis of cell surface markers (phenotypic characterization). Cell suspension was also used for magnetic bead-based separation of a single cell population for gene expression analysis (phenotypic characterization).

Fibroblasts and macrophages constitute major cell populations of the solid tumor stroma, exert pro-tumorigenic functions, and hold considerable potential as therapeutic targets (20). Thus, we aimed to develop 3D tumorigenic spheroids using NSCLC cells, fibroblasts, and monocytes with a potential interest to mimic the natural counterpart. To understand the role of tumor microenvironment in cellular heterogeneity and plasticity, we have also made non-tumorigenic spheroids combining lung epithelial cells with fibroblasts and monocytes in order to compare with tumorigenic spheroids. Briefly, NSCLC cell lines (A549 and NCI-H460 lung adenocarcinoma) or lung epithelial cell line BEAS-2B were cultured in combination with MRC-5 lung embryonic fibroblasts, and THP-1 monocytes in the DMEM media supplemented with 10% FBS and hence named as A549+MRC-5+THP-1 (AMT spheroids) and NCI-H460+MRC-5+THP-1 (HMT spheroids), and BEAS-2B+MRC-5+THP-1 (BMT spheroids) (**Figures 1A–C**). We provided diverse cell counts for spheroid formation across literature and started with three different cell counts per spheroid, i.e., 5000, 8000, and 10,000. To define each cell type’s ratio in the spheroid, we used different ratios of tumor cells: fibroblasts: monocytes, i.e., 1:1:1, 2:2:1, 4:2:1, and 5:2:1 with increasing concentrations of tumor cells and maintenance of CAF density near 40% as reported in the previous literature (21). After optimizing the cell count (10,000 cells per drop), ratio (tumor/normal cells: fibroblasts: monocytes = 5:4:1), and method (hanging drop) (**Figures 1D–F** and **Figure S1**), the spheroids were allowed to grow undisturbed for d4, d7, and d10. Developed spheroids were used for the physicochemical and functional characterization by immunofluorescence analysis, whole-mount analysis, and single-cell dissociation for gene expression analysis (**Figure 1G**).

### Characterization of Multicellular 3D Spheroid’s Morphology, Viability, and Cell Proliferation

**Figure 2:**
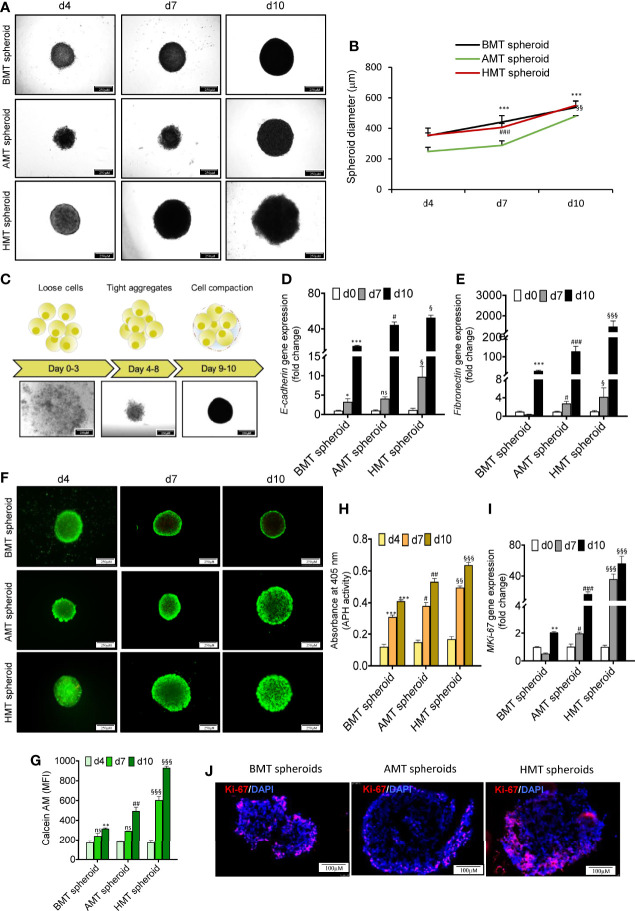
Phenotypic characterization of multicellular 3D spheroid’s morphology, viability, and cell proliferation. **(A)** Microscopic images of multicellular 3D spheroids composed of BEAS-2B, MRC-5, and THP-1 (BMT spheroid); A549, MRC-5, and THP-1 (AMT spheroid); and NCI-H460, MRC-5, and THP-1 (HMT spheroid) on day 4, day 7, and day 10. **(B)** Measurement of spheroid’s diameter on day 4, day 7, and day 10. **(C)** Schematic diagram (upper panel) and microscopic images (lower panel) of spheroid development at different time points displaying initial loose association of cells (days 0-3) followed by tight aggregates (days 4-8) leading to compact spheroid formation (day 9onwards). **(D, E)** RT-qPCR analysis of *E-cadherin* **(D)** and *fibronectin* **(E)** mRNA levels in BMT, AMT, and HMT spheroids on day 0, day 7, and day 10. **(F, G)**Calcein-AM and propidium iodide (PI) staining **(F)**, and the quantification of Calcein-AM-stained live cells **(G)** in BMT, AMT, and HMT spheroids on day 4, day 7, and day 10(n=3). **(H)** Determination of acid phosphatase(APH) activity in developed spheroids(n=3). **(I)** RT-qPCR analysis of cell proliferation marker *MKi67* in BMT, AMT, and HMT spheroids on day 4, day 7, and day 10(n=3). **(J)** Immunofluorescence images of *Ki67* protein in day 10 spheroids section. DAPI was used for nuclei counterstaining. GAPDH is a loading control for RT-qPCR studies. Fold change for *E-cadherin*, *fibronectin*, and *MKi-67*gene expression was calculated keeping the spheroid cell cocktail (BEAS-2B/MRC-5/THP-1, A549/MRC-5/THP-1, and NCI-H460/MRC-5/THP-1) at day 0 as control. All experiments were performed in triplicate (n=3). Data represented as mean ± S.D. 2D vs BMT over time *p< 0.05, **p< 0.01, ***p< 0.001; BMT vs AMT over time ^#^p< 0.05, ^##^p< 0.01, ^###^p< 0.001; BMT vs HMT over time ^§^p< 0.05, ^§§^p< 0.01, ^§§§^ p< 0.001; ns, non-significant.

Multicellular 3D spheroid (AMT, HMT, and BMT) diameters were measured after days 4, 7, and 10. We observed an increasing trend in spheroid size and diameters with increasing days **(** **Figures 2A, B** **).** BMT and HMT spheroids were 355 ± 3.21 and 347 ± 2.5 μm in diameter on day 4, 425 ± 5 and 405 ± 3.21 μm on day 7, and 514 ± 12.16 and 551 ± 2.8 μm on day 10. AMT spheroids were initially of smaller size as compared to BMT and HMT spheroids, with a diameter of 263 ± 3.6 μm on day 4, 289 ± 3.6 μm on day 7, and 489 ± 2.5 μm on day 10 (**Figures 2A, B**). Moreover, multicellular AMT, HMT, and BMT spheroids formed were of uniform size and diameter, as displayed by microscopic images **(** **Figure S2A** **)**. The morphology of multicellular spheroids largely depends on cell types and culture methods. Based on cell compactness, spheroid morphology can be categorized as loose aggregates, tight aggregates, or compact spheroids **(** **Figure 2C** **)**. Loose aggregates have low cell-cell or cell-matrix interactions and are easy to disintegrate, whereas cells are tightly bound to each other in rigid spheroids. The cell adhesion proteins, such as E-cadherin and fibronectin, play a critical role in cell spheroid formation (18). We noticed a marked induction of E-cadherin and fibronectin gene expression with an increase in the number of days in spheroid formation **(** **Figures 2D, E** **)**. Spheroids were also analysed for N-cadherin expression to test whether this spheroid formation is associated with epithelial-mesenchymal transition (EMT). We found a notable downregulation of N-cadherin expression in AMT and HMT spheroids as compared to BMT spheroids on day 7 and day 10 **(** **Figure S2B** **)** suggesting spheroid rigidity. Next, to check the cellular viability within tumor spheroids, calcein-AM/PI staining was performed **(** **Figures 2F, G** **)**, suggesting cell growth and proliferation of these spheroids over time. We have also determined the cell viability by measuring cytosolic acid phosphatases (APH) activity **(** **Figure 2H** **).** However, to confirm cellular proliferation potential within these spheroids, we measured the MKi67 cell proliferation marker gene and Ki-67 protein expression by RT-qPCR and immunostaining, respectively. MKi67 is a nuclear protein that is present during all active phases of the cell cycle (G1, S, G2, and M) but is absent in resting cells (G0) (22). As shown in **Figure 2I**, a significant increase in MKi67 gene expression was noticed with an increase in days of all three spheroids. However, tumorigenic 3D spheroids showed a much higher cell proliferation rate than non-tumorigenic spheroids. It was also evident from immunofluorescence staining of MKi-67 levels in day 10 tumorigenic spheroids compared to non-tumorigenic spheroids **(** **Figure 2J** **)**.Moreover, we have sorted fibroblast and macrophage populations from tumorigenic spheroids **(** **Figures S3A–D**) and analyzed MKi-67 proliferation marker in them. An increasing trend of *MKi-67* gene expression was observed in fibroblasts at day 10 whereas induction was noticed in isolated macrophages from day 7 onwards in tumorigenic spheroids **(** **Figures S3E, F** **)**.

### Assessment of TME Features in Multicellular 3D Spheroids

**Figure 3:**
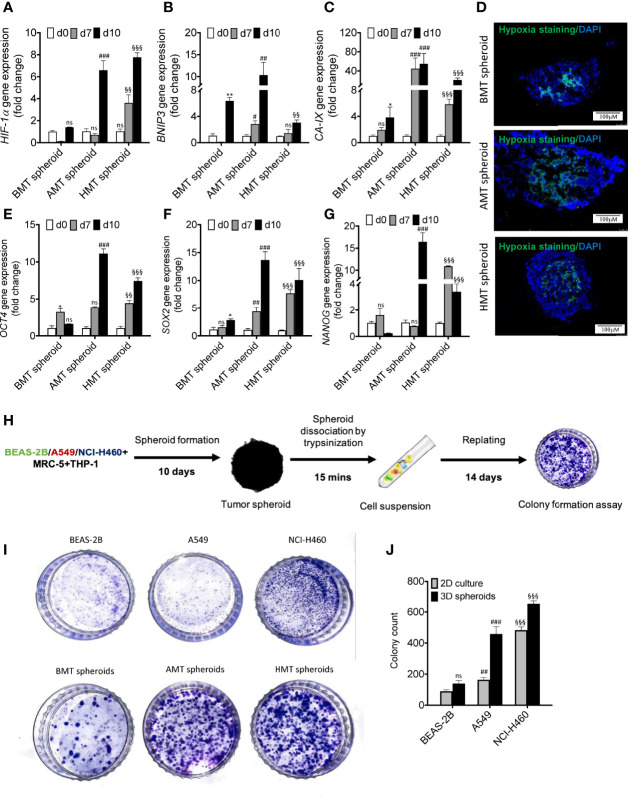
Assessment of TME features in multicellular 3D spheroids. **(A–C)** RT-qPCR analysis of hypoxia-regulated gene expressions such as *HIF-1α* **(A)** *BNIP3* **(B)** and *CA-IX* **(C)** in BMT, AMT, and HMT spheroids on day 0, day 7, and day 10. **(D)** Image-iTGreen hypoxia stainingin BMT, AMT, and HMT spheroid sections (6 micron). DAPI was used for nuclei counterstaining. **(E–G)** RT-qPCR analysis of pluripotency markers gene expressions such as *OCT4* **(E)**, *SOX2* **(F)**, and *NANOG* **(G)** in BMT, AMT, and HMT spheroids on day 0, day 7, and day 10. **(H)** Schematic diagram representing methodology adopted for colony formation assay using multicellular spheroids. **(I, J)** Clonogenic assay was performed with BEAS-2B, A549, and NCI-H460 cell lines and with cancer cell suspension of BMT, AMT, and HMT spheroids, photographed and quantified **(J)** GAPDH was used as loading control for RT-qPCR analysis. Fold change was calculated keeping the 2D cells at day 0 as control. We compared the gene expression profiles over time in each spheroid separately. All experiments were performed in triplicates (n=3). Data represented as mean ± S.D. 2D vs BMT over time *p< 0.05, **p< 0.01; BMT vs AMT over time #p< 0.05, ^##^p< 0.01, ^###^p< 0.001; BMT vs HMT over time ^§§^p< 0.01, ^§§§^p< 0.001; ns, non-significant.

Hypoxic zone and necrotic areas are major characteristics of solid tumors (23). Cancer and stromal cells activate survival signalling pathways in response to rapid oxygen depletion and avascularity (24). Hypoxia in the tumor microenvironment induces stable expression of hypoxia-inducible factors (HIF), which promotes angiogenesis *via* VEGF induction (23, 25), stimulates pluripotency by increased expression of pluripotent markers (*OCT4*, *SOX2*, *NANOG*) (25, 26), and increases acidosis through enhanced expression of carbonic anhydrase IX (*CA-IX*) (27). We found a significant increase in the expression of hypoxia-related genes such as *HIF-1α*, *BNIP3*, and *CA-IX* with an increase in the number of days of tumor spheroids development as compared to non-tumorigenic spheroids **(** **Figures 3A–C** **)**. This was also evident in isolated cell population of fibroblasts and macrophages from tumorigenic spheroids **(** **Figures S3G–J** **)**. The tumorigenic 3D spheroids exhibited a prominent hypoxic zone, as indicated by the increased Image-iT Green hypoxia staining **(** **Figure 3D** **)**. Moreover, a significant enhancement of induced pluripotent stem cell (iPSC) markers (*OCT4*, *SOX2*, and *NANOG*) gene expression was observed in tumorigenic 3D spheroids with increasing the number of days as compared to non-tumorigenic spheroids **(** **Figures 3E–G** **)** indicating manifestation of pluripotency stimulation in tumorigenic 3D spheroids. The gene expression pattern of these iPSC markers in the isolated fibroblasts and macrophages are not in consistent with the data of tumorigenic spheroids **(** **Figures S3K–P** **)** indicating non-stemness nature of the isolated fibroblasts and macrophage cell populations. Further, tumor spheroid-derived cells more aggressively form the colony than the cancer cells in 2D culture **(** **Figures 3H–J** **)**. These results suggested the development of pathophysiologically relevant TME features within the tumorigenic 3D spheroids.

### Alteration of Fibroblast and Monocyte Cell Plasticity in Tumorigenic Multicellular 3D Spheroids

**Figure 4:**
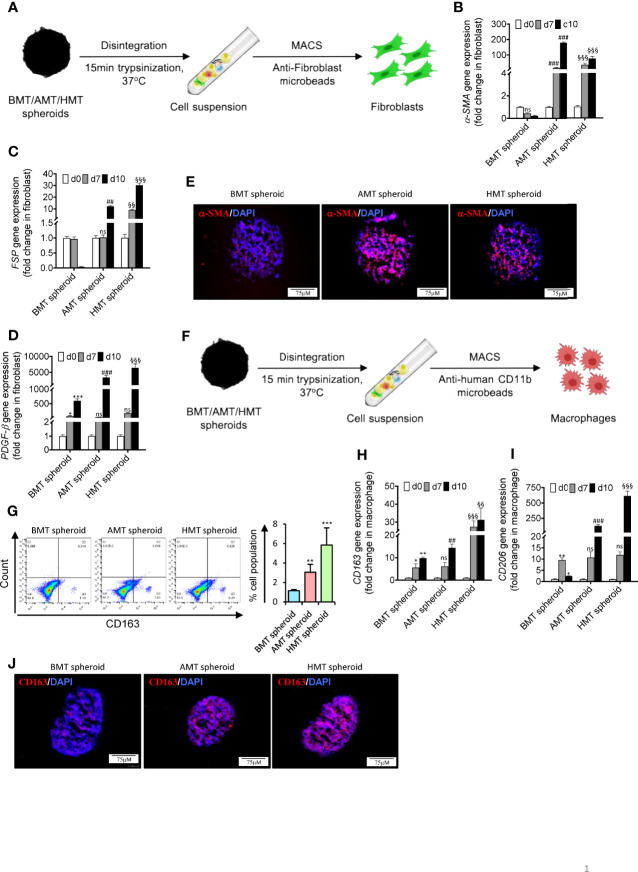
Fibroblast activation and monocyte TAM polarization in tumorigenic multicellular 3D spheroids. **(A)** Schematic diagram representing isolation of fibroblasts from 3D multicellular spheroids using anti-fibroblast microbeads-based cell sorting to examine CAF markers such as α-SMA, FSP/S100A4, and PDGF-β. **(B–D)** RT-qPCR analysis shows a relative abundance of α-SMA **(B)**, FSP **(C)**, and PDGF-β **(D)** mRNA levels in isolated fibroblasts of BMT, AMT, and HMT spheroids on day 0, day 7, and day 10. **(E)** Immunofluorescence staining of α-SMA in BMT, AMT, and HMT spheroid sections (6 micron). DAPI was used for nuclei counterstaining. **(F)** Schematic diagram representing isolation of macrophages from 3D multicellular spheroids using anti-human CD11b microbeads to examine TAM markers CD163 and CD206. **(G)** Flow cytometric analysis and quantification of CD163+ cells in BMT, AMT, and HMT spheroids on day 10. **(H, I)** RT-qPCR analysis of CD206 **(H)** and CD163 **(I)** gene expression in macrophages isolated from BMT, AMT, and HMT spheroids on day 0, day 7, and day 10. **(J)** Immunofluorescence staining of CD163 in BMT, AMT, and HMT spheroid sections (6 micron). DAPI was used for nuclei counterstaining. GAPDH is used as a loading control for RT-qPCR analysis. Fold change for CAF markers was calculated keeping MRC5 as control cells and fold change for TAM population was calculated keeping THP-1 cells as control cells. All experiments were performed in triplicate (n=3). Data represented as mean ± S.D. 2D vs BMT over time *p< 0.05, **p< 0.01, ***p< 0.001; BMT vs AMT over time ^##^p< 0.01, ^###^p< 0.001; BMT vs HMT over time ^§§^p< 0.01, ^§§§^p< 0.001; ns, non-significant.

Fibroblasts and macrophages are the major stromal and immune cell populations, respectively, present within the TME of lung cancer. Fibroblasts are usually found in an inactive form in normal tissues and regulate proper tissue architecture by controlling ECM composition (28). However, when in direct or indirect contact (through secretory proteins) with tumor cells, fibroblasts get activated to multiple subtypes of cancer-associated fibroblasts (CAFs) expressing a unique repertoire of different genes, including collagens and elastins (28, 29). We have isolated fibroblasts from individual 3D spheroids using anti-fibroblast magnetic microbeads (**Figure 4A** **)** and examined the gene expression pattern of CAF-specific markers. A significant induction of CAF-specific markers gene expression such as α-SMA (**Figure 4B** **)**, FSP (**Figure 4C** **)**, and PDGF-β (**Figure 4D**) was observed in the isolated fibroblasts obtained from day 7 and day 10 of AMT and HMT spheroids as compared to BMT spheroids. Moreover, AMT and HMT spheroids also exhibited an increased level of α-SMA as compared to BMT spheroids (**Figure 4E**). CAF activation in tumorigenic 3D spheroid was further validated with human lung tissue biopsies obtained from non-cancerous and lung adenocarcinoma patients. Increased expression of α-SMA (**Figure S4A**), and FSP (**Figure S4B**) were evident in lung adenocarcinoma samples as compared to non-cancerous tissue samples.

Like CAFs, macrophages residing within the TME adopt a characteristic feature of the CD163+CD206+ tumor-associated macrophage (TAM) phenotype (2, 17,). To assess the model’s capability of recapitulating the immune contexture of TME, such as differentiation of monocytic THP-1 cells into myeloid M2-like TAM phenotype, we have isolated the monocytes from the spheroids **Figure 4F** and found a striking induction of CD163 (scavenger receptor for the hemoglobin-haptoglobin complex) and CD206 expression (**Figures 4G–I**) in the isolated macrophages obtained from day 7 and day 10 of AMT and HMT spheroids as compared to BMT spheroids. Furthermore, AMT and HMT spheroids also exhibited an increased level of CD163 as compared to BMT spheroids (**Figure 4J**). This data suggests that A549/NCI-H460 lung cancer cells in spheroids promote the differentiation of monocytic cells towards M2-like TAM macrophages. This was further validated in human non-cancerous and lung adenocarcinoma biopsies where lung adenocarcinoma samples showed higher expression of CD163 and CD206 (**Figures S4E, F**) as compared to the non-cancerous samples.

### Macrophage Showing Endothelial Markers in Tumorigenic Multicellular 3D Spheroids

**Figure 5:**
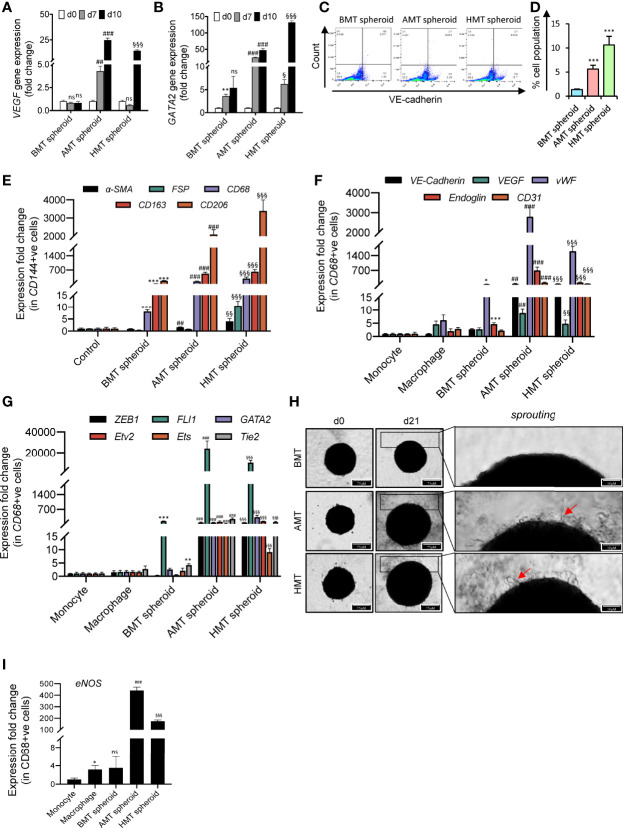
Angiogenic potential of malignant 3D multicellular spheroids. **(A, B)** RT-qPCR analysis of endothelial cell markers gene expression such as *VEGF* **(A)** and *GATA2* **(B)** in BMT, AMT, and HMT spheroids on day 0, day 7, and day 10. **(C, D)** Flow cytometric analysis **(C)** and quantification **(D)** of VE-cadherin+ cells in BMT, AMT, and HMT spheroids on day 10. **(E)** RT-qPCR analysis of *a-SMA*, *FSP*, *CD68*, *CD163*, and *CD206* mRNA levels in CD144+ cells isolated from BMT, AMT, and HMT spheroids on day 14. **(F, G)** RT-qPCR analysis of endothelial cell markers (*VE-cadherin, VEGF, vWF, Endoglin, and CD31*) **(F)** and angiogenic regulators (*ZEB1, FLI1, GATA2, Etv2, Ets, and Tie2*) gene expression **(G)** in THP-1 macrophages and CD68+ cells, respectively, isolated from BMT, AMT, and HMT spheroids on day 14h. **(H)**. Representative images of BMT, AMT, and HMT spheroids on day 21 after placing the spheroid of day 14 on a collagen matrix and maintained in endothelial cell media for 7 days. Cell sprouting (red arrow) were detected in tumor spheroids. **(I)** RT-qPCR analysis of *eNOS* gene expression in CD68+ cells of these spheroids on day 21. GAPDH was used as loading control for RT-qPCR analysis. Fold change for CD144+ cells was calculated keeping the 2Dcells(control)at day 0as control and fold change for CD68+ cells was calculated keeping THP-1 monocytes (2D) as control. All experiments were performed in triplicates (n = 3). Data represented as mean ± S.D. 2D vs BMT over time *p< 0.05, **p< 0.01, ***p< 0.001; BMT vs AMT over time ^##^p< 0.01, ^###^p< 0.001; BMT vs HMT over time ^§^p< 0.05, ^§§^p< 0.01, ^§§§^p< 0.001; ns, non-significant.

In recent years, significant strides have been made to investigate tumor angiogenesis in 3D tumor spheroids. It caused a paradigm shift in our understanding of how TME influences angiogenic signatures. Interestingly, we have found a massive induction of vascular endothelial growth factors (VEGF) and GATA-2 gene expression in tumorigenic spheroids over time compared to non-tumorigenic spheroids **(** **Figures 5A, B** **)**. Both VEGF and GATA2 are considered pivotal factors governing endothelial cell development (30, 31). We next sought to analyze the VE-cadherin (CD144) positive (endothelial-specific marker) cells in these spheroids. To our surprise, we noticed a significant number of CD144+ cell population in the tumorigenic spheroids. FACS analysis revealed that 5.61% of cells in the AMT spheroids and 10.63% of cells in the HMT spheroids were CD144+ on the day 10 of these spheroid’s development as compared to non-tumorigenic spheroids (**Figures 5C, D**) indicating the appearance of endothelial cell characteristics in these malignant 3D spheroids. To explore the cell type that gained endothelial features, we have sorted out CD144+ cells from these 3D multicellular spheroids on day 14 and checked the expression profile of fibroblast markers (α-SMA, and FSP) and macrophage markers (CD68, CD163, and CD206). Increased abundance of macrophage markers in CD144+ cells as compared to fibroblast markers (**Figure 5E**) indicated that M2 polarized macrophages are the cell source within the tumor spheroid that could be involved in endothelial cell formation. However, to further examine the potency of these M2 polarised macrophages on angiogenic stimulation, we sorted the CD68+ (pan-macrophage marker) cell population from the spheroids and examined the gene expression profile of different endothelial cells markers and angiogenic regulators. We noticed a profound induction of various endothelial cell marker gene expressions such as *VE-Cadherin*, *VEGF*, *vWF*, *CD31*, and *endoglin* (**Figure 5F**), along with the enhancement of gene expression for diverse angiogenic transcription factors like *Etv2*, *FLI1*, *GATA2*, *ZEB1*, *Tie2*, and *ETS* (**Figure 5G**). Moreover, the tumorigenic spheroids displayed cell sprouting when day 14 spheroids were placed on the collagen matrix for seven days (**Figure 5H**) with profound induction of endothelial nitric oxide synthase (*eNOS*) gene expression (**Figure 5I**). All these results indicated that macrophages within the tumor microenvironment may gain endothelial characteristics and can exhibit features of tumor endothelial cells in the later stages of cancer.

### Validation of Tumorigenic 3D Spheroid features in Zebrafish Xenograft Model

**Figure 6:**
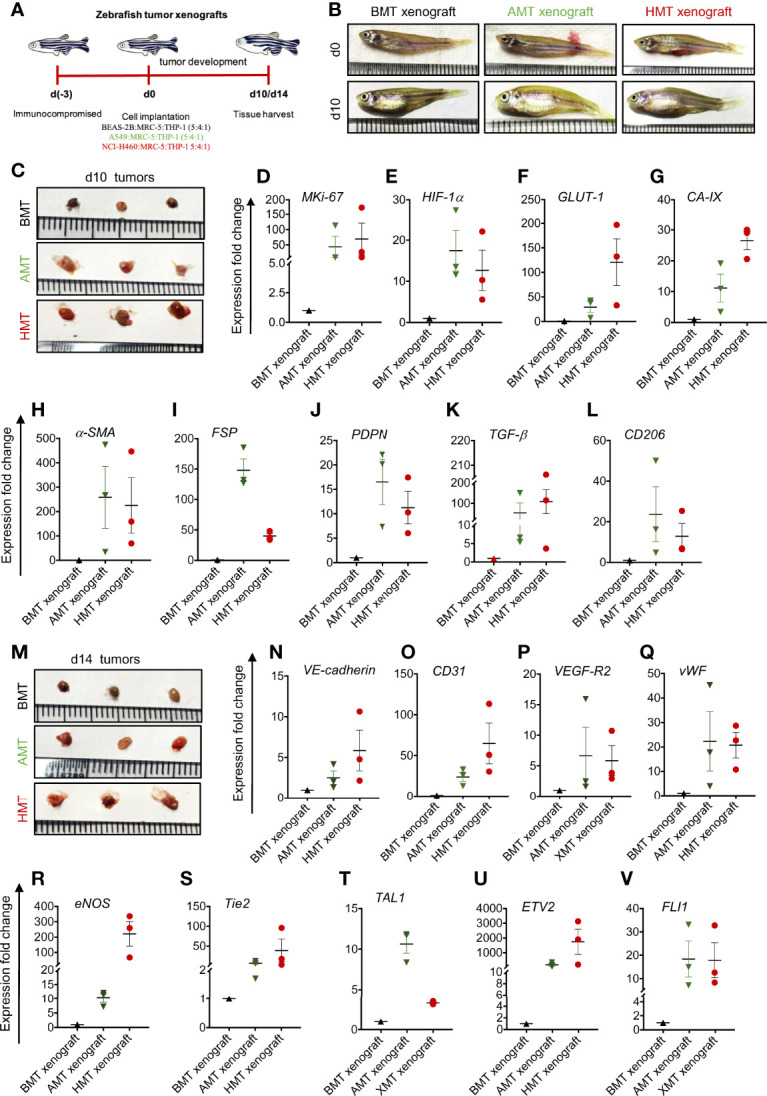
Validation of 3D tumor spheroid data in zebrafish xenograft model. **(A)** Schematic diagram representing experimental design indicating days on which zebrafish was immunocompromised [d(-3)], mixed cell population was injected into the peritoneal cavity [d(0)], and BMT, AMT, and HMT xenografts were collected on d10 and d14. **(B)** Representative photographs of zebrafish on d0, and d10 of tumor development. **(C)** Representative photographs of zebrafish BMT, AMT, and HMT tumor xenografts (n=3) on day 10. **(D-L)** RT-qPCR analysis of different gene expression in BMT xenograft (black, n=3), AMT xenograft (green, n=3), and HMT xenograft (red, n=3) on day 10. **(D)** *MKi-67* proliferation marker; **(E–G)** hypoxia related markers- *HIF-1α* **(E)**, *GLUT-1* **(F)**, and *CA-IX* **(G)**; **(H–K)** cancer associated fibroblast markers- *α-SMA* **(H)**, *FSP* **(I)**, *PDPN* **(J*)*** and *TGF-β* **(K)**; **(L)** tumor associated macrophage marker *CD206*; **(M)** Representative photographs of zebrafish BMT, AMT, and HMT xenografts (n=3) on day 14. **(N–V)** RT-qPCR analysis of different gene expression in these xenografts on day 14. **(N–R)** endothelial cell markers- *VE-cadherin* **(N)**, *CD31* **(O)**, *VEGF-R2* **(P)**, *vWF* **(Q)**, *eNOS* **(R)**; **(S–V)** angiogenic regulators *Tie2* **(S)**, *TAL1* **(T)**, *ETV2* **(U)**, *FLI1* **(V)**. GAPDH was used as loading control for RT-qPCR analysis. All experiments were performed in triplicate (n=3).

To validate the results obtained from 3D spheroids, we have developed a zebrafish tumor xenograft model by intraperitoneal delivery of the mixed cell population using the same number and ratio as described previously in the spheroid development protocol. A mixed cell population (total cell number 10,000) in 5:4:1 (BEAS-2B/A549/NCI-H460: MRC-5: THP-1) ratio was injected into the peritoneum of immuno-compromised adult zebrafish (**Figures 6A, B**), xenografts were isolated on day 10 and day 14 (**Figures 6C–M**), and the cell population was subjected to gene expression analysis of different markers. Similar to 3D spheroids, we noticed increased expression of the MKi67 proliferation marker in day 10 tumorigenic xenografts compared to non-tumorigenic xenografts (**Figure 6D**). Next, we analyzed the expression pattern of hypoxia-specific marker *HIF-1α* and its regulated genes such as *GLUT-1* and *CA-IX*. Both AMT and HMT tumorigenic xenografts showed enhanced expression of HIF-1α, GLUT-1, and CA-IX than BMT non-tumorigenic xenografts (**Figure 6E–G**) indicating the development of profound hypoxic zones within day 10 of tumorigenic xenograft development. To check the fate of normal fibroblasts within these xenografts, we have examined the expression profile of CAF markers α-SMA, FSP, PDPN, and TGF-β. The AMT and HMT xenografts showed a marked induction of all these CAF markers (**Figures 6H–K**) compared to BMT xenografts. Moreover, we have also checked the plasticity of THP-1 monocytes in xenografts. Similar to the 3D tumorigenic spheroids, AMT/HMT xenografts exhibited profound induction of TAM population as evidenced by the CD206 expression marker (**Figure 6L**). Similar to the results of endothelial-like cell formation in tumor spheroids mentioned above, we analyzed the gene expression of various endothelial (**Figures 6N–R**) and angiogenic transcription factors (**Figures 6S–V**) in the day 14 xenografts. Results indicated the presence of endothelial-like cell characteristics in these tumorigenic xenografts. All these results suggest the pathophysiological state of AMT/HMT xenografts and show alteration of cellular plasticity in the tumor xenografts model.

## Discussion

The tumor microenvironment (TME) comprises tumor cells and supporting stromal cells, including fibroblasts, different immune cells and their subtypes, which are critical for cancer disease progression and sustainability (20, 32). TME-originated molecular cues are responsible for altering phenotypic features of tumor resident cells and supporting tumor growth and progression (2, 32). Similar to the *in-vivo* TME condition, the external microenvironment of *in-vitro* cell cultures also influences their gene expression pattern, metabolic requirements, and signalling pathways (33). For instance, co-culture of cancer cells with CAFs leads to the promotion of stem-cell-like properties in squamous cell carcinoma (34), cancer cells and their exosomes derived TGF-β augmented proliferation and expression of CAF markers (35), and the co-culture of pancreatic cancer cells with monocytes and fibroblasts induces the production of immunosuppressive cytokines which are known to promote polarization towards M2 like macrophages and myeloid-derived suppressive cells (MDSCs) (36). All these reports highlighted the importance of developing *in-vitro* tumorigenic 3D cultures to imitate the *in-vivo* TME features and establish tumor-stroma interaction.

To develop a multicellular spheroid model that could mimic the *in-vivo* TME features of solid tumors, we have optimized the cell numbers and ratio of tumor cells, fibroblasts, and monocytes. Multicellular spheroids are usually formed by techniques that would allow cell suspension to grow in low adhesion conditions (anchorage-independent manner), ranging from the rotatory system (37); the liquid overlay method (38); and uncoated ultra-low attachment U-shaped plates (39). In the present study, we optimized the modified hanging drop method protocol to develop multicellular tumorigenic 3D spheroids. These heterogeneous spheroids, combining lung adenocarcinoma cells with fibroblasts and monocytes, remarkably shaped the tumor ecosystem and represented a physiologically relevant cancer research model. The main advantage of this model is one can efficiently create the TME features and study the cellular crosstalk between different cell types and their role in various pathophysiological changes associated with cancer development and progression. To understand the role of TME in cellular heterogeneity and plasticity, we have also made non-tumorigenic spheroids with lung epithelial cells with fibroblasts and monocytes. This method has previously been used to prepare tumor spheroids using hepatocellular carcinoma, breast cancer, and pancreatic cancer cell lines (40–42). Most of these 3D spheroid studies are particularly focused on therapeutic applications of different small molecules on cancer cell death, with limited experimental analysis of tumor heterogeneity or mechanistic study of TME on tumor aggressiveness and metastasis. Single-cell transcriptomic analysis has unveiled the functional heterogeneity in the multicellular 3D spheroids of breast cancer (43) and cutaneous melanoma (44), highlighting different immunomodulators and pathways activated in these spheroids. However, the cellular heterogeneity and plasticity in non-small cell lung cancer (NSCLC) have not been explored due to the lack of a relevant multicellular 3D lung cancer spheroid model. Since the prognosis of NSCLC is very poor, it would be worth developing a suitable model that could simulate the lung cancer TME heterogeneity for a better understanding of NSCLC development and progression. Therefore, the *in-vitro* study of 3D NSCLC spheroids TME is a new outlook in lung cancer research.

In this study, we found the generation of hypoxic core, induction of pluripotency, fibroblasts activation, and macrophage plasticity in our multicellular A549 or NCI-H460 tumorigenic 3D spheroid models (AMT or HMT spheroids) which display characteristic features of TME in NSCLC (23, 25). As it grows over time, both AMT and HMT spheroids exhibit striking induction of hypoxia as indicated by the upregulation of HIF-1α expression, a master regulator of tissue hypoxia, and its regulated genes such as *BNIP3* and *CA-IX*. Our multicellular 3D spheroids displayed uniformity with excellent cell proliferative capacity as indicated by APH activity and MKi-67 proliferation marker expression. The rate of cell proliferation at late time points is relatively high in AMT and HMT spheroids compared to BMT spheroids. Moreover, time-dependent enhancement of E-cadherin and fibronectin gene expression confirms these spheroids’ integrity.

The NSCLC spheroids also demonstrated an upregulation of pluripotency marker expressions such as OCT4, SOX2, and NANOG and induction of tumorigenicity as evidenced by colony formation assay. The developed NSCLC spheroid is of immense value as it exhibits conversion of normal fibroblasts to CAFs and differentiation of THP-1 monocytes to TAM phenotypic population, as indicated by the increase of CAFs and TAM markers expression, similar to the TME of different solid tumors. Surprisingly, we noticed induction of endothelial characteristics in the macrophage population of tumorigenic 3D spheroids at the late time point, as indicated by the appearance of endothelial cell markers and angiogenic regulators gene expression. This observation suggests that the TME of NSCLC spheroids may involve in the lineage conversion of the macrophage population towards tumor endothelial cells (TECs). However, the nature of molecular cue(s) and underlying molecular mechanism(s) of TAM to TEC conversion are not explicit. Therefore future studies in this direction may identify the novel target(s) for anti-angiostatic cancer therapeutics. It is interesting to note that both AMT and HMT spheroids exhibited similar TME characteristic features, including alteration of cellular plasticity. Data obtained from these spheroids are reproducible in the *in-vivo* zebrafish tumor xenograft model and human NSCLC tissue samples suggesting their physiological validation. Hence, tumorigenic multicellular 3D spheroids represent an excellent tool for the scientific community working on tumor heterogeneity and cellular plasticity in the TME of NSCLC. Moreover, this 3D spheroid model would also be a boon for researchers working in tumor regenerative medicine and those who do not have access to human samples.

In conclusion, the present study successfully established a multicellular 3D NSCLC model that simulates the *in-vivo* TME features, allowing us to study fibroblast activation and macrophage plasticity. These physiologically relevant 3D tumor spheroids would be a promising tool for studying NSCLC tumor biology and drug efficacy.

## Materials and Methods

### Reagents and Antibodies

Please refer to **Supplementary Table 1**: Key resources table for details of resources used for this study.

### Cell Culture

Human non-small cell lung cancer cell lines (A549 and NCI-H460) and human monocytic THP-1 cell lines were procured from the National Centre for Cell Science (NCCS) Pune, India. Cells were cultured in RPMI1640 media supplemented with 10% FBS and 1% Penicillin-Streptomycin at 37°C in a humidified chamber with 5% CO2. MRC-5 human lung fibroblast cells were obtained from the ATCC, USA; and BEAS-2B human bronchial epithelial cells was a generous gift from Prof. Anita K. Verma. MRC-5 and BEAS-2B were cultured in EMEM and LHC-9 media, respectively, supplemented with 10% FBS and 1% Penicillin-Streptomycin solution at 37°C in a humidified chamber with 5% CO2.

### Multicellular 3D Spheroid Formation

The multicellular tumorigenic and non-tumorigenic 3D spheroids were prepared using the hanging drop method. The detailed protocol of the 3D multicellular spheroid development is given in **Figure 1**. Briefly, lung epithelial cells (BEAS-2B) or lung adenocarcinoma cells (A549 and NCI-H460) were combined with MRC-5 lung fibroblasts and THP-1 monocytes in a complete growth medium (DMEM, 10% FBS and 1% Penicillin/Streptomycin). Developed spheroids are named BMT (BEAS-2B+MRC5+THP-1), AMT (A549+MRC5+THP-1), and HMT (NCI-H460+MRC5+THP-1). Cell suspension was prepared using BEAS-2B/A549/NCI-H460 cells in combination with MRC-5 and THP-1 in a 5:4:1 ratio, i.e., 5000:4000:1000 cells/ml and 25μl (cell suspension in 5:4:1 ratio and complete DMEM medium)droplets were spotted onto 90mm cell culture dish lid. We have standardised 25μl as maximum volume of cell suspension to prepare a droplet as it will not fall down while inverting the lids. Approximately 50 droplets can be placed on each 90mm dish. The lid was then gently placed on a plate without disturbing the droplets, and 6 ml of autoclaved water was added to the dish’s bottom to keep the cells hydrated. Plates were maintained at 37°C in a humidified incubator with 5% CO_2_ for 4 days to allow the spheroid formation. After day 4, spheroids were routinely monitored under the microscope and imaged to examine cell aggregation and proliferation. For a live/dead assay, five spheroids each were collected in an eppendorf tube on day 4, day 7, and day 10, gently washed with PBS and incubated with 1μM Calcein-AM and 2 mg/ml propidium iodide solution for 10 min. On termination of the incubation period, spheroids were gently washed with PBS twice, pipette out on a glass dish using 50μl tips, and imaged using an inverted fluorescent microscope (Leica DMi8, Germany).

### Human Samples

Human lung tissue biopsies were collected from the patients with or without non-small cell lung cancer (adenocarcinoma) from the Postgraduate Institute of Medical Education and Research (PGIMER). The study protocol was approved by the Institute Ethics Committee (IEC), PGIMER, Chandigarh. The Declaration of Helsinki protocols were followed and informed written consent was obtained from all the patients. Demographic details of the patients are mentioned in **Supplementary Table 2**.

### Zebrafish Xenograft Tumor Development

Adult zebrafish (Danio ratio) of both sexes were maintained under a 14 h/10 h day/night photoperiod at 28 ± 2°C in 4L fish tanks containing autoclaved, sterilized water supplemented with penicillin-streptomycin. Water was changed every day and fish were fed twice a day with commercially available feed (MicroMac, Aqua World, India). The Zebrafish lung cancer xenograft model was developed following a previously described method with slight modifications (45). Briefly, caerulomycin (100 mg/Kg body weight) was administered in the intraperitoneal region of zebrafish for immunosuppression, 3 days prior to cell transplantation and fish were maintained in water containing 1% penicillin-streptomycin. On day 3, 10^4^ cells/fish [5 (BEAS-2B/A549/NCI-H460): 4 (MRC-5): 1 (THP-1)] were suspended in PBS and injected into the peritoneal cavity of the zebrafish using a 5μl Hamilton syringe (Hamilton, Nevada USA). Subsequently, fish were maintained in distilled water with 1% penicillin-streptomycin for the next 10 or 14 days allowing for tumor xenograft development. On day 10 and day 14, fish were sacrificed, tumor tissue was harvested and used for downstream experiments. The protocol and procedures were approved by the Institutional Animal Ethics Committee, Kirori Mal College, University of Delhi (Protocol no: DU/KR/IAEC/ZF2021/1).

### Acid Phosphatase Assay

Acid phosphatase (APH) activity of the spheroids was determined following the method described by Friedrich et al (46), using 4-nitrophenyl phosphate as a substrate. Briefly, spheroids were washed with PBS and placed in a 96-well plate (10 spheroids/well). APH assay buffer (100 µl), containing para-Nitrophenyl phosphate (PNPP, 2 mg/ml), Triton-X (0.1%) in Citrate buffer (0.1M), was added, and the plates were incubated for 90 min at 37°C. On termination of incubation, NaOH (1M, 10 µl) was added to each well, and absorbance was measured at 405 nm on a microplate reader (Multiskan GO Microplate Spectrophotometer, Thermo Fisher Scientific, Finland).

### Quantitative Real Time PCR

Total RNA was extracted from the spheroids, isolated cells, human tissue, and zebrafish xenograft samples using Trizol reagent according to the manufacturer’s instructions. 100 spheroids per condition were collected in an eppendorf, tube, gently washed with PBS, disintegrated using 200μl of 0.25% trypsin-EDTA solution for 15 min at 37°C, and centrifuged at 1200 rpm for 5 min at 4°C to proceed further for RNA isolation. RNA quality was measured using NanoDrop One/One Microvolume UV-Vis spectrophotometer (Thermo Fisher Scientific, USA) and treated with DNase I. For mRNA expression analysis, cDNA was prepared from 500ng of total RNA using the iScript cDNA synthesis kit, following the manufacturer’s guidelines. Single-cell RNA isolation was performed using a single-cell lysis kit following the manufacturer’s protocol. 100ng of total RNA was reverse transcribed using SuperScript VILO cDNA synthesis kit. PowerUp SYBR^®^ Green Master Mix qPCR (2X) Universal was used to perform RT-qPCR analysis in a Quant-Studio 5 Real-Time PCR System (Applied Biosystem, USA) to quantify the relative mRNA expression level using gene-specific primers. After the final extension, a melt curve analysis was performed to ensure the specificity of the products. Data were normalized to the expression of the GAPDH reference gene. Primer sequences used for RT-qPCR are listed in **Supplementary Table 3**.

### Immunofluorescence Staining

Immunostaining was performed on spheroid cryosections using specific antibodies. Briefly, spheroids were washed with PBS, fixed with 4% paraformaldehyde for 15 mins, and embedded in 1% agarose solution (20 µl agarose droplet was used to coat each spheroid. The spheroids can further be made visible in OCT by colouring agarose solution with ponceau stain. Spheroids in agarose droplets were then embedded in -30°C cryofixed, in OCT, and kept at -80°C before further use. OCT-embedded spheroids were cryosectioned (6 microns), fixed with cold-acetone, blocked with 5% Bovine Serum Albumin (BSA), and incubated overnight with specific primary antibodies. Signal was visualized by subsequent incubation with fluorescence-tagged appropriate secondary antibodies (Alexa 594-tagged anti-mouse, 1:1,000 dilution; Alexa 594-tagged anti-rabbit, 1:1,000 dilution) and counter-stained with DAPI. Images were captured by a fluorescence microscope (Leica DMi8, Germany), and analysis was performed using LASX software.

### Colony Formation Assay

To determine the clonogenicity of multicellular 3D spheroids, day 10 spheroids were disintegrated using 0.25% trypsin-EDTA solution for 15 min, centrifuged at 1200 rpm for 5 min at 4°C, and cell pellets were washed and resuspended with complete DMEM medium. Cell suspension was then seeded at a density of 4000 cells/60mm dish and allowed to grow for the next 14 days. To determine the clonogenicity of BEAS-2B, A549 and NCI-H460 cells in 2D culture, cells (4000 cells/60 mm dish) were seeded and continued to grow for 14 days. On day 14, culture media was removed, and cells were washed with PBS twice and fixed with 4% paraformaldehyde for 15 min. After fixation, cells were stained with 0.5% crystal violet staining solution at room temperature for 20 min, and then plates were rinsed with distilled water, air-dried, and photographed.

### Flow Cytometry

Spheroids were disintegrated using 0.25% trypsin-EDTA solution at 37°C for 15 min, centrifuged at 1200 rpm for 5 min at 4°C and cell pellets were washed with chilled PBS. Cell pellets were then re-suspended in cell staining buffer (PBS pH 7.4 + 0.2% FBS+ 0.09% NaNO_3_) and blocked with Fcγ blocker (TruStainFcX™, Biolegend). Cells were incubated with PE conjugated VE-Cadherin (anti-human), APC conjugated CD163 (anti-human), APC conjugated α-SMA (anti-human), APC conjugated CD68 (anti-human) antibodies for 1 h in rotatory mixer at 4°C. Labelled cells were washed twice with PBS, re-suspended in cell staining buffer and analyzed in a Flow cytometer (BD Accuri C6+, BD Biosciences, San Jose, CA) using FlowJo™ v10.6.1 software.

### Magnetic Cell Sorting

For positive selection of single-cell using magnetic beads, approximately 100 BMT, AMT, and HMT spheroids were taken per condition in an eppendorf tube. Spheroids were washed once with PBS and disintegrated using 0.25% trypsin-EDTA solution at 37°C for 15 min, centrifuged at 1000 rpm for 5 min at 4°C, counted, and 10^7^ cells of each spheroid were resuspended in 80μl of a cold buffer containing PBS, 0.5% BSA, and 2mM EDTA. CD11b/CD68, VE-cadherin, and anti-fibroblast magnetic bead separation (Miltenyi Biotech) were done according to the protocol provided by the manufacturer. Briefly, 20μl/10^7^ of the respective antibodies were added per condition to isolate CD11b, VE-cadherin, and α-SMA positive cell population, mixed well, and incubated for 30 mins at room temperature. Cells were washed by adding 1ml of cold buffer, centrifuged at 1000 rpm for 5 mins, the supernatant was aspirated, and the cells were resuspended in 500μl buffer. For magnetic separation, the MACS column was prepared by rinsing with 3ml buffer, the cell suspension was applied to the column, and flow-through containing unlabelled cells was collected. After washing the column 3 times with 3ml buffer, the column was removed from the separator and placed on a collection tube. Magnetically labeled cells were collected by adding 5ml buffer and by firmly pushing the plunger into the column to get the desired cell population. Flow-through was collected to obtain tumor cell population, centrifuged, and used for further analysis. CD68+ cells were isolated using protein G magnetic beads. Protein G magnetic beads were tagged with specific antibodies for 2 hours at 4°C. Disintegrated spheroids were incubated with the tagged magnetic beads for 2 hours at 4°C, centrifuged at 1000 rpm for 5 minutes to remove excess antibodies, resuspended in PBS, and positive cells were isolated by magnetic separation.

### Statistical Analysis

All data analyses were performed using GraphPad Prism software (v.8.0; GraphPad Software, Inc., La Jolla, CA, USA). Data represented as mean ± S.D. Students *t*-test determined statistical significance, and *p* value indicated significance. Data represent as mean ± S.D. A level of *p*< 0.05 was considered significant.

## Data Availability Statement

The original contributions presented in the study are included in the article/**Supplementary Material**. Further inquiries can be directed to the corresponding author.

## Ethics Statement

The studies involving human participants were reviewed and approved by The Institute Ethics Committee (IEC), PGIMER, Chandigarh, The patients/participants provided their written informed consent to participate in this study. The animal study was reviewed and approved by Institutional Animal Ethics Committee, Kirori Mal College, University of Delhi (Protocol no: DU/KR/IAEC/ZF2021/1).

## Author Contributions

Study concept and design: LA, and DP. Acquisition of data: LA, MK, AV and NS. Analysis and interpretation of data: LA, SD, AV, and DP. Drafting of the manuscript: LA, SD, and DP. Critical revision of the manuscript: LA, MK, SD, NS, AV, and DP. Study supervision: DP. All authors read and approved the final manuscript.

## Funding

This work was supported by the SERB- Women Excellence Award Project, India (SB/WEA-02/2017) and the SERB-EarlyCareer Research Award Project, India (ECR/2017/000892) to DP. Financial support from IIT Ropar in the form of ISIRD Grant and Bio-Consortium Grant is gratefully acknowledged.

## Conflict of Interest

The authors declare that the research was conducted in the absence of any commercial or financial relationships that could be construed as a potential conflict of interest.

## Publisher’s Note

All claims expressed in this article are solely those of the authors and do not necessarily represent those of their affiliated organizations, or those of the publisher, the editors and the reviewers. Any product that may be evaluated in this article, or claim that may be made by its manufacturer, is not guaranteed or endorsed by the publisher.

## Supplementary Material

Supplementary Figure 1 Optimization of the protocol for spheroid development using different cell concentrations i.e. 5,000, 8,000 and 10,000 per drop using modified hanging drop method and different ratios of tumor/normal cells: fibroblasts: monocytes (1:1:1, 2:2:1, 4:2:1 and 5:2:1) were used for proper spheroid development.

Supplementary Figure 2
**(A)** Microscopic images of BMT, AMT, and HMT spheroids showing spheroid uniformity at day 10. **(B) **RT-qPCR analysis of *N-cadherin* gene expression in BMT, AMT, and HMT spheroids on day 7, and day 10. GAPDH was used as loading control. Fold change was calculated keeping the 2D cells at day 0 as control. All experiments were performed in triplicate (n=3). Data represented as mean ± S.D. BMT vs AMT over time ^###^p< 0.001; BMT vs HMT over time ^##^p< 0.01, _§§§_ p< 0.001; ns, non-significant.

Supplementary Figure 3
**(A-B)** Percentage of α-SMA+ cells (fibroblasts) isolated from AMT and HMT spheroids using anti-fibroblast human microbeads. **(C, D)** Percentage of CD68+ cells (macrophages) isolated from AMT and HMT spheroids using anti-human CD11b microbeads. **(E–P) **RT-qPCR analysis of MKi-67 proliferation marker **(E, F)**, *HIF-1α* **(G, I)**, *CA-IX* **(H, J)**, pluripotency markers *OCT4* **(K, N)**, *SOX2* **(L, O)**, and *NANOG* **(M, P)** in α-SMA+ cells or CD68+ cells isolated from AMT, and HMT spheroids on day 7, and day 10. Fold change was calculated keeping MRC5 or THP-1 as control cells/day 0. All experiments were performed in triplicates (n=3). Data represent as mean ± S.D. *p< 0.05, **p< 0.01, ***p< 0.001; ns, non-significant.

Supplementary Figure 4
**(A–B)**. Images representing H&E staining of non-cancerous normal lung tissue biopsies and cancerous lung adenocarcinoma biopsies. **(A, C)**. RT-qPCR analysis showing *α-SMA* **(C)**, and *FSP1* **(D)** gene expression in normal lung tissue biopsies (non-cancerous, n=5) and lung adenocarcinoma tissue biopsies (cancerous, n=5). **(E, F)**. RT-qPCR analysis showing *CD163* **(E)**, and *CD206* **(F)** gene expression in normal lung tissue biopsies (non-cancerous, n=5), and lung adenocarcinoma tissue biopsies (cancerous, n=5).

## Abbreviations

TME, tumor microenvironment; NSCLC, non-small-cell lung cancer; HIF-1α, hypoxia inducible factor-1α; ECM, extracellular matrix; CAFs, cancer-associated fibroblasts; TAMs, tumor-associated macrophages; TECs, tumor endothelial cells; RT-qPCR, real-time quantitative PCR; 3D, 3 dimensional.
