# Supplementary material for: Development of a Multicellular 3D Tumor Model to Study Cellular Heterogeneity and Plasticity in NSCLC Tumor Microenvironment
Source: Front Oncol. 2022 Jun 28;12:881207. doi: 10.3389/fonc.2022.881207 (PMC9273950; doi:10.3389/fonc.2022.881207)

Supplementary figure 1 (Fig S1):

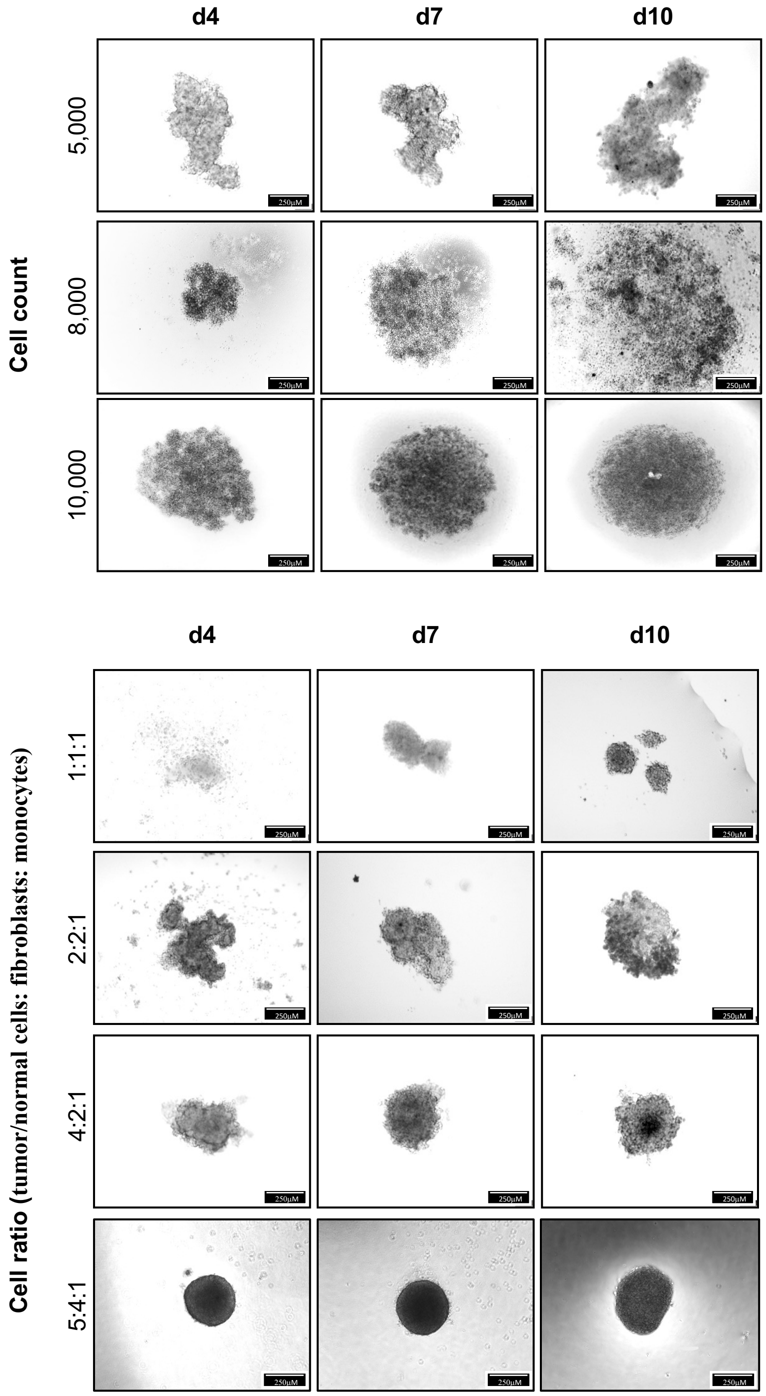

Supplementary figure 2 (Fig. S2):

a.

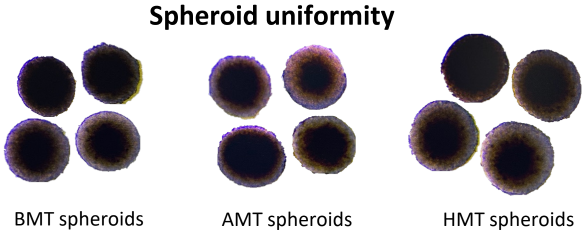

b.

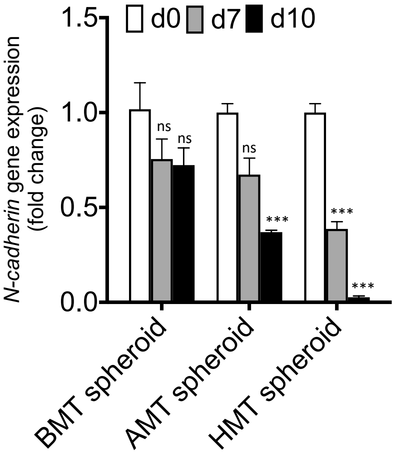

Supplementary figure 3 (Fig. S3):

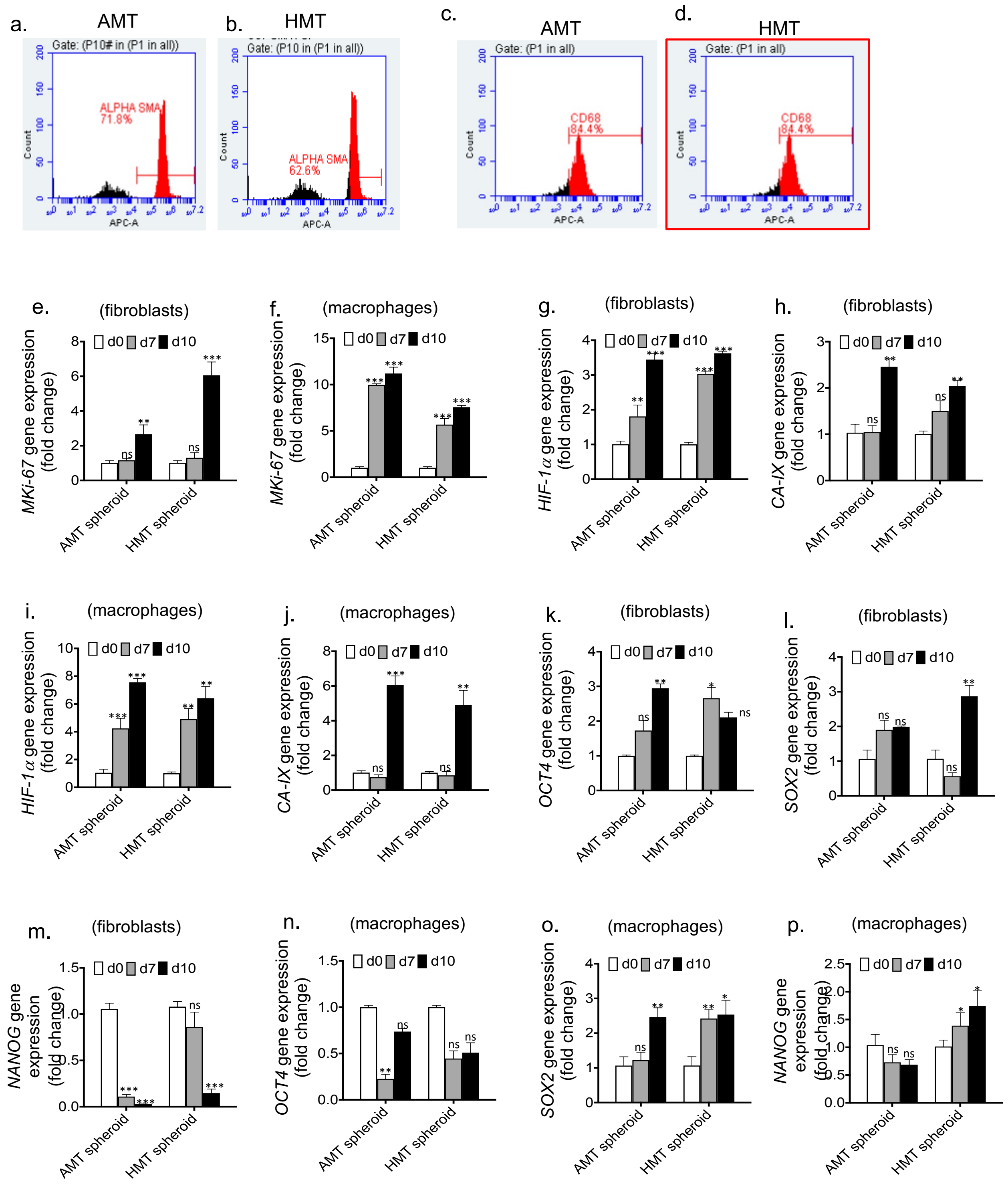

Supplementary figure 4 (Fig. S4):

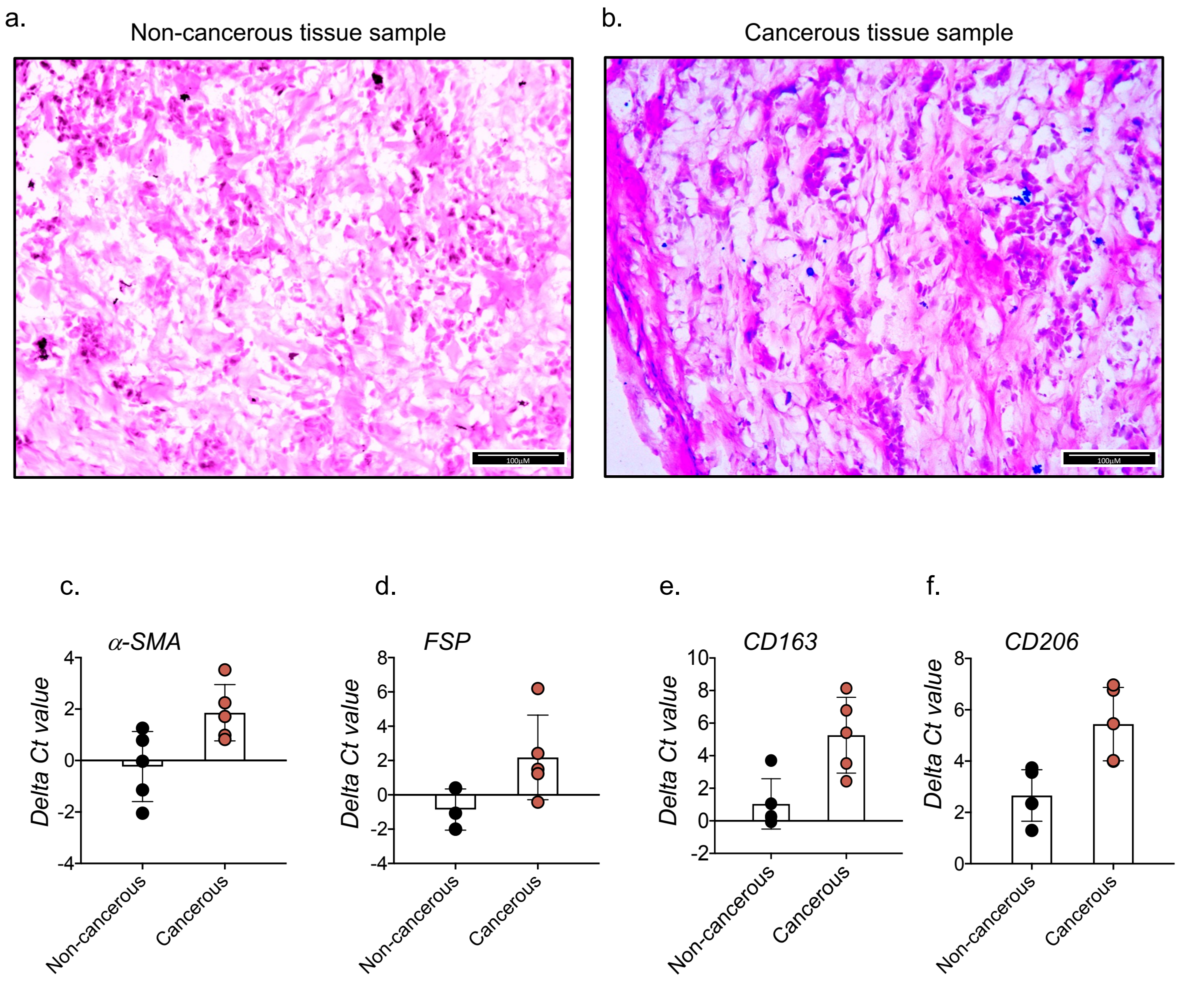

Supplement: Supplementary Figure 1 — Optimization of the protocol for spheroid development using different cell concentrations i.e. 5,000, 8,000 and 10,000 per drop using modified hanging drop method and different ratios of tumor/normal cells: fibroblasts: monocytes (1:1:1, 2:2:1, 4:2:1 and 5:2:1) were used for proper spheroid development. [file Presentation_1.pdf]
