# Supplementary material for: Development of a Multicellular 3D Tumor Model to Study Cellular Heterogeneity and Plasticity in NSCLC Tumor Microenvironment
Source: Front Oncol. 2022 Jun 28;12:881207. doi: 10.3389/fonc.2022.881207 (PMC9273950; doi:10.3389/fonc.2022.881207)
Supplement: Supplementary file 2 [file Table_1.pdf]

Supplementary tables:

Supplementary Table 1: KEY RESOURCES

REAGENT OR RESOURCE	SOURCE	IDENTIFIER
**Antibodies**		
anti-Ki-67	Thermo fisher scientific	Cat# MA5-14520
anti-HIF-1α	Thermo fisher scientific	Cat# PA1-16601
anti-α-SMA	Cell Signaling Technology	Cat# 48938
anti-CD163	Abcam	Cat# 182422
anti-CD68	Abcam	Cat# 125212
anti-VE-Cadherin	Cell Signaling Technology	Cat# 2158
anti-Mouse IgG Alexa fluor 594	Invitrogen	Cat# A11032
anti-Rabbit IgG Alexa fluor 594	Cell Signaling Technology	Cat# 8889
APC CD163	eBioscience	Cat# 17-1639-42
PE anti-human CD144	Biolegend	Cat# 348505
APC anti-human CD68	Biolegend	Cat# 333809
APC anti-human α-SMA	R&D systems	Cat# IC1420A
Anti-human CD11b microbeads	Miltenyi Biotec	Cat# 130-049-601
Anti-human CD144 microbeads	Miltenyi Biotec	Cat# 130-097-857
Anti-fibroblast microbeads	Miltenyi Biotec	Cat# 130-050-601
**Chemicals**		
DMEM	Gibco	Cat# 11995073
RPMI 1640	Gibco	Cat# 11875093
EMEM	ATCC	Cat# 30-2003
Endothelial cell growth media	Cell Biologics, USA	Cat# H1168
LHC-9 media	Gibco	Cat# 12680013
FBS	Gibco	Cat# 10082147
Penicillin/Streptomycin	Gibco	Cat# 15140122
DPBS	Gibco	Cat# 14190-144
Trypsin 0.25% EDTA	Gibco	Cat# 25200072
Collagen I, Rat tail	Gibco	Cat# A1048301
Calcein-AM	Thermo fisher scientific	Cat# C3099
Propidium iodide	Thermo fisher scientific	Cat# P1304MP
DAPI	Thermo fisher scientific	Cat# D1306

Trizol reagent	Invitrogen	Cat# 15596018
Image-iT Green hypoxia reagent	Invitrogen	Cat# I14834
iScript cDNA synthesis kit	BIO-RAD	Cat# 1708891
Universal SYBR Green Supermix	BIO-RAD	Cat# 172-5124
Single Cell Lysis Kit	Thermo Fisher Scientific	Cat# 4458235
SuperScript VILO cDNA synthesis kit	Thermo Fisher Scientific	Cat# 11754-050
Crystal violet stain	Sigma	Cat# C6158
Protein G Magnetic beads	BIO-RAD	Cat# 161-4023
16% Formaldehyde Solution	Thermo fisher scientific	Cat# 28906
Tissue freezing medium	Leica	14020108926
Triton X-100	Sigma	Cat# T8787
Ethanol	Merck	1009832500
Isopropanol	Himedia	Cat# MB063
Acetone	Merck	Cat# SD7F670190
Chloroform	SRL	Cat# 84155
Methanol	Merck	Cat# SGOP700408
BSA	Sigma	A2153
PMA	Sigma	P8139
DPX Mountant	SRL	Cat# 88147
4-Nitrophenyl Phosphate	TCI	N1109

Supplementary Table 2:

**Clinicopathological features**		
**LUAD patients**		
Number of patients	5	
Age (median range)	50 years (38-75 years)	
Gender	2 (Male)	
	3 (Female)	
Smoking history	1 (Smoker)	
	4 (Non-smoker)	
Histological subtype	Lung adenocarcinoma	
TNM staging	Stage 4 (Metastatic)	
**Non-cancerous patients**		**Disease category**
Number of patients	5	Tuberculosis, Granulomatous inflammation
Age (median range)	54 years (24-75 years)	
Gender	2 (Male)	
	3 (Female)	
Smoking history	1 (Smoker)	
	4 (Non-smoker)	

Supplementary Table 3:

Gene name	Forward primer (5'-3')	Reverse primer (5'-3')
E-cadherin	TCGGGAGGGTGAATCTCAGG	GAAGAAGAGCAAGCAATAGCAG
Fibronectin	ACAACACCGAGGTGACTGAGAC	GGACACAACGATGCTTCCTGAG
MKi-67	GAAAGAGTGGCAACCTGCCTTC	GCACCAAGTTTTACTACATCTGCC
HIF-1α	GTGGTGGTTACTCAGCACT	CGTCCCTCAACCTCTCAGTT
BNIP3	TCAGCATGAGGAACACGAGCGT	GAGGTTGTCAGACGCCTTCCAA
CA-IX	GTGCCTATGAGCAGTTGCTGTC	AAGTAGCGGCTGAAGTCAGAGG
GLUT-1	TTGCAGGCTTCTCCAACTGGAC	CAGAACCAGGAGCACAGTGAAG
OCT4	CCTGAAGCAGAAGAGGATCACC	AAAGCGGCAGATGGTCGTTTGG
SOX2	GCTACAGCATGATGCAGGACCA	TCTGCGAGCTGGTCATGGAGTT
NANOG	CTCCAACATCCTGAACCTCAGC	CGTCACACCATTGCTATTCTTCG
α-SMA	CTATGCCTCTGGACGCACAACT	CAGATCCAGACGCATGATGGCA
FSP	CAGAACTAAAGGAGCTGCTGACC	CTTGGAAGTCCACCTCGTTGTC
PDGF-β	GCCATCAGCAGCAAGGCGAG	GCAGGTCAGAACGAAGGTGC
TGF-β	ACTGCGGATCTCTGTGTCAT	AGTAGTGTTCCCCACTGGTC
PDPN	GTGCCGAAGATGATGTGGTGAC	GGACTGTGCTTTCTGAAGTTGGC
CD163	AAAGCGAAGACAGAGACAGC	TCATGGGAATTTTCTGAGGAAT
CD206	ACGATCCGACCCTTCCTTGA	GCTTGCAGTATGTCTCCGCT
VEGF	TTGCCTTGCTGCTCTACCTCCA	GATGGCAGTAGCTGCGCTGATA
VEGF-R2	GGAACCTCACTATCCGCAGAGT	CCAAGTTCGTCTTTTCCTGGGC
GATA2	TAGAGCCCTGTAGTTCCTGCC	ACTGGGGGTTGGGATGGTTC

eNOS	CAACAGCATCTCCTGCTCAGA	CGAACACACAGAACCTGAGGG
VE-cadherin	GAAGCCTCTGATTGGCACAGTG	TTTTGTGACTCGGAAGAACTGGC
N-cadherin	CCTCCAGAGTTTACTGCCATGAC	GTAGGATCTCCGCCACTGATTC
vWF	GTGTGTCCGAGTGAAGGAGG	CAGCACGCTGAGGTCTTACA
Endoglin	CCCACAAGTCTTGCAGAAACA	CTGGCTAGTGGTATATGTCACCT
CD31	AAGTGGAGTCCAGCCGCATATC	ATGGAGCAGGACAGGTTCAGTC
ZEB1	TACCAGAGGATGACCTGCCA	TGCCCTTCCTTTCCTGTGTC
FLI1	ACGGAAGTGCTGTTGTCACACC	CAAGCTCCTCTTCTGACTGAGTC
ETS	TGAGGTAGCTTAGAGATGTAGCG	ATGTGCCAGCATCAGCTACT
Tie2	TGCCACCCTGGTTTTTACGG	TTGGAAGCGATCACACATCTC
ETV2	ACGTCTCGGAAAATTCCCCC	ATGTCTCTGCTGTCGCTGTC
TAL1	CTTAGCCAGCCGCTCGCCTC	TGTCTGGTGGGTGGGTGGGG
GAPDH	CCCTTCATTGACCTCAACTACA	ATGACAAGCTTCCCGTTCTC
